# RIPK1 is a critical modulator of both tonic and TLR-responsive inflammatory and cell death pathways in human macrophage differentiation

**DOI:** 10.1038/s41419-018-1053-4

**Published:** 2018-09-24

**Authors:** Julian Buchrieser, Maria Jose Oliva-Martin, Michael D. Moore, Joshua C. D. Long, Sally A. Cowley, Jose Antonio Perez-Simón, William James, Jose Luis Venero

**Affiliations:** 1 Sir William Dunn School of Pathology, University of Oxford, Oxford, OX1 3RE UK; 2 Instituto de Biomedicina de Sevilla-Hospital Universitario Virgen del Rocío/CSIC/Universidad de Sevilla, Sevilla, 41012 Spain; 3 Departamento de Bioquímica y Biología Molecular, Facultad de Farmacia, Universidad de Sevilla, Sevilla, Spain; 4 Present Address: Institut Pasteur, 28 rue du Dr Roux, 75015 Paris, France

## Abstract

In this study, we took advantage of human-induced pluripotent stem cells (hiPSC) and CRISPR/Cas9 technology to investigate the potential roles of RIPK1 in regulating hematopoiesis and macrophage differentiation, proinflammatory activation, and cell death pathways. Knock-out of RIPK1 in hiPSCs demonstrated that this protein is not required for erythro-myeloid differentiation. Using a well-established macrophage differentiation protocol, knock-out of RIPK1 did not block the differentiation of iPSC-derived macrophages, which displayed a similar phenotype to WT hiPSC-derived macrophages. However, knock-out of RIPK1 leads to a TNFα-dependent apoptotic death of differentiated hiPSC-derived macrophages (iPS-MΦ) and progressive loss of iPS-MΦ production irrespective of external pro-inflammatory stimuli. Live video analysis demonstrated that TLR3/4 activation of RIPK1 KO hiPSC-derived macrophages triggered TRIF and RIPK3-dependent necroptosis irrespective of caspase-8 activation. In contrast, TLR3/4 activation of WT macrophages-induced necroptosis only when caspases were inhibited, confirming the modulating effect of RIPK1 on RIPK3-mediated necroptosis through the FADD, Caspase-8 pathway. Activation of these inflammatory pathways required RIPK3 kinase activity while RIPK1 was dispensable. However, loss of RIPK1 sensitizes macrophages to activate RIPK3 in response to inflammatory stimuli, thereby exacerbating a potentially pathological inflammatory response. Taken together, these results reveal that RIPK1 has an important role in regulating the potent inflammatory pathways in authentic human macrophages that are poised to respond to external stimuli. Consequently, RIPK1 activity might be a valid target in the development of novel therapies for chronic inflammatory diseases.

## Introduction

Macrophages are key cells of the innate immune system. They are distributed throughout the tissues of the body, and play a key role in host defense, tissue homeostasis, and development^1^. Macrophages must constantly strike a balance between resting homeostatic functions, activated pro-inflammatory functions and cell death^2^. Too little activation can lead to poor pathogen clearance; too much activation can lead to inflammation-mediated pathologies^3^. Similar considerations apply to cell death; too little cell death in the context of intracellular infection of macrophages can lead to pathogen spread while too much cell death can prevent the cells from performing their effector function^4^. These pathways have been shown to share finely regulated signaling platforms, in which receptor-interacting serine/threonine-protein kinase 1 (RIPK1) plays an essential role^5–8^. RIPK1 has been reported to shift the balance between cell survival, apoptosis, and necroptosis upon TNFα stimulation. Initially, it was reported to act as a kinase in the formation of the “necrosome” and triggering of RIPK3-dependent necroptosis^9,10^. However, a kinase-independent role for RIPK1 was later described, which suggests a scaffolding role for RIPK1 to inhibit caspase-8-dependent apoptosis and, paradoxically, necroptosis^11,12^. Although the dual function of RIPK1 is best understood in the context of TNFα signaling, a wide range of other triggers, such as IFNαR, TLRs, viral infection, and genotoxic stress have recently been described to trigger RIPK1 activation and necrosome formation^13^. Furthermore, RIPK1 has also been shown to play a role in the induction of pro-inflammatory gene expression independently of cell death^5,6,14^.

Consistent with its role in regulating inflammation and cell death, the scaffolding role of RIPK1 has also been observed to be required for normal development. For example, the knock-out (KO) of RIPK1 in mice results in perinatal death due to systemic inflammation in the absence of infection^15–17^, whereas mice with kinase inactive RIPK1 can develop normally^18–20^. Further characterization of the RIPK1 KO mouse model showed that the deletion of RIPK1 led to bone marrow aplasia and loss of hematopoietic stem and progenitor cells (HSPCs)^16^. In a follow-up study, two conditional RIPK1 KO mice were generated, in which RIPK1 was deleted from adult mice or from hematopoietic cells. For both models, the same loss of hematopoietic cells was observed accompanied by an increase in pro-inflammatory cytokines, which was hypothesized to cause the death of HSPCs through a RIPK3*-*dependent mechanism^17,21^. Interestingly, when cultured ex vivo, RIPK1 KO hematopoietic stem cells remained viable and differentiated normally, supporting the view that the loss of hematopoietic progenitors in RIPK1 KO mice is a consequence of a RIPK3-dependent systemic inflammation. However, the poor engraftment of RIPK1 KO progenitor cells in immunocompromised mice, which were outcompeted by wild-type progenitors, suggests that there may be underlying factors beyond systemic inflammation^21^. Interestingly, recently two independent groups identified Z-DNA-binding protein 1 (ZBP1), a RHIM-containing protein, as a critical component in inducing MLKL-induced necroptosis, whose activity is kept under control by RIPK1 through its RHIM domain^22,23^. ZBP1 was originally identified as a pathogen sensor and has been shown to elicit a wide array of immune-related functions^24^.

Given the challenges of distinguishing the cell-intrinsic from the systemic inflammatory effects of RIPK1 KO in whole-animal models, there are advantages to using cell differentiation and culture models, in which cellular environments can be controlled more precisely. Moreover, the availability of human-induced pluripotent stem cells (hiPSC) bypasses the need to study a model organism that may differ in important ways from the human. Accordingly, we have exploited recent advances in human iPS-derived in vitro myelopoeisis^25,26^, which produces consistently large numbers of macrop hage precursors that can be further differentiated to microgliaor other tissue macrophages, according to context^27,28^. By using CRISPR/Cas9 technology, we knocked-out RIPK1 in hiPSCs to study its role in human macrophage development and function in the absence of confounding factors.

Here, we report that RIPK1 KO in human hiPSCs does not affect undifferentiated hiPSCs or the initial differentiation of hiPSC-derived macrophage precursors, but leads to a TNFα-dependent death of fully differentiated hiPSC-derived macrophages (iPS-MΦ) and progressive loss of iPS-MΦ production irrespective of external pro-inflammatory stimuli. In addition, RIPK1 KO iPS-MΦ expressed higher basal levels of pro-inflammatory cytokine transcripts than wild-type cells. The absence of RIPK1 only increased the sensitivity of iPS-MΦ to TNFα-induced cell death very modestly. On the other hand, RIPK1 KO iPS-MΦ were particularly sensitive to caspase-8 independent, TLR3 and TLR4-mediated TRIF-RIPK3-dependent cell death and RIPK3-dependent inflammatory responses.

## Results

### KO of RIPK1 in hiPSCs does not impair hematopoietic differentiation

**Fig. 1:**
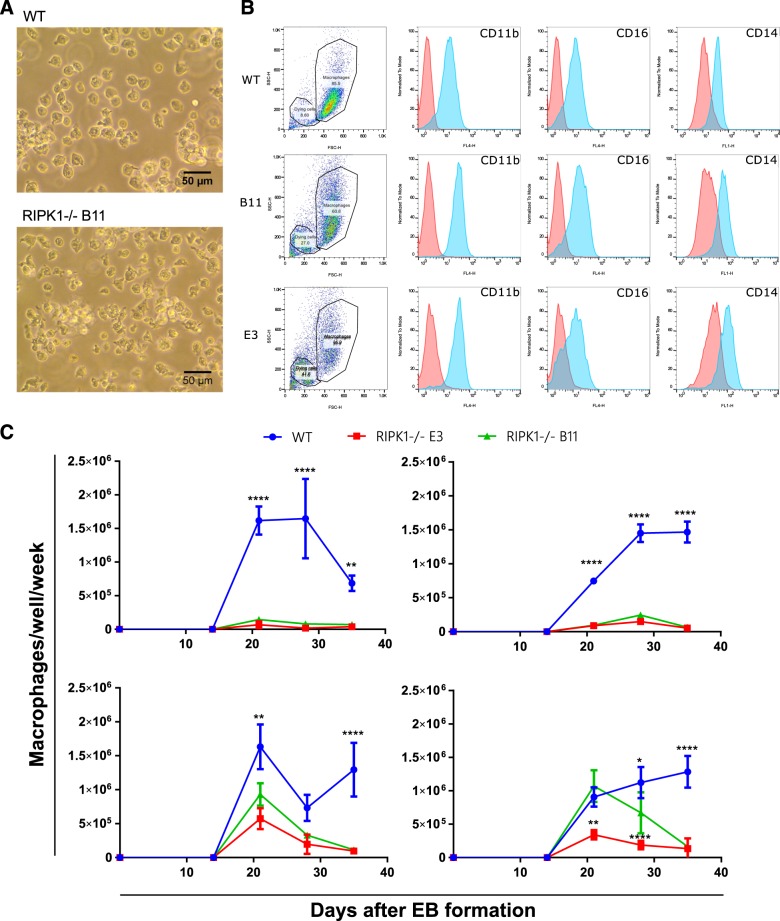
RIPK1^−/−^ iPSCs can differentiate into macrophages but RIPK1 deficiency results in a progressive loss of macrophage production over time. **a** Representative images of iPSC-MΦ early in differentiation, before harvest. No visual difference was observed between WT and RIPK1 KO iPSC-MΦ at this early stage. **b** Flow cytometry staining of iPSC-derived macrophages for CD11b, CD16, and CD14 myeloid surface makers, showing live cell gate on the left and histogram plots on the right, antibody staining (blue) and isotype (red). **c** Noncumulative production of iPSC-derived macrophages per well over a period of 35 days of four independent experiments. Each time point represents the mean number of iPSC-derived macrophages harvested per well of WT (*n* = 3), RIPK1^-/-^ B11 (*n* = 3), and RIPK1^-/-^ E3 (*n* = 3). Error bars denote SD. Statistical comparisons were done using two-way ANOVA

We used CRISPR/Cas9 technology to introduce frame-shift mutations in exon 5 of RIPK1 and thereby knock out functional expression of the gene (Supplementary Figure 1). Two RIPK1 homozygous KO hiPSC lines were tested alongside a wild-type control clone. RIPK1 KO hiPSC lines cells were viable, karyotypically normal and showed no signs of differentiation (Supplementary Figure 2). RIPK1 expression was completely knocked-out at the protein level in both RIPK1 KO hiPSC lines as shown by western blot (Supplementary Figure 4B and C). Furthermore, all hiPSC lines were capable of forming embryoid bodies (EBs), with no significant differences in size and morphology between EBs from both WT and RIPK1 KO clones (Supplementary Figure 3). RIPK1 KO hiPSCs were differentiated to macrophages using a well-established macrophage differentiation protocol^25,26^. WT and RIPK1 KO iPS-MΦ had an indistinguishable morphology and phenotype based on key macrophage surface markers (Fig. 1a and b). Residual RIPK1 mRNA (potentially encoding a truncated and non-functional polypeptide) was knocked-down by over 10-fold in RIPK1 KO iPS-MΦ (Supplementary Figure 4A). However, from week 2 onwards, wild-type EBs generated a substantial number of iPS-MΦ, as expected, while both RIPK1 KO clones exhibited an initial production between week 2 and 4 post-EB formation, followed by a rapid loss of macrophage production (Fig. 1c). The initial production of RIPK1 KO iPS-MΦ was variable from experiment to experiment, and was significantly lower than in WT hiPSCs.

### Hematopoietic progenitors are viable and differentiate normally

**Fig. 2:**
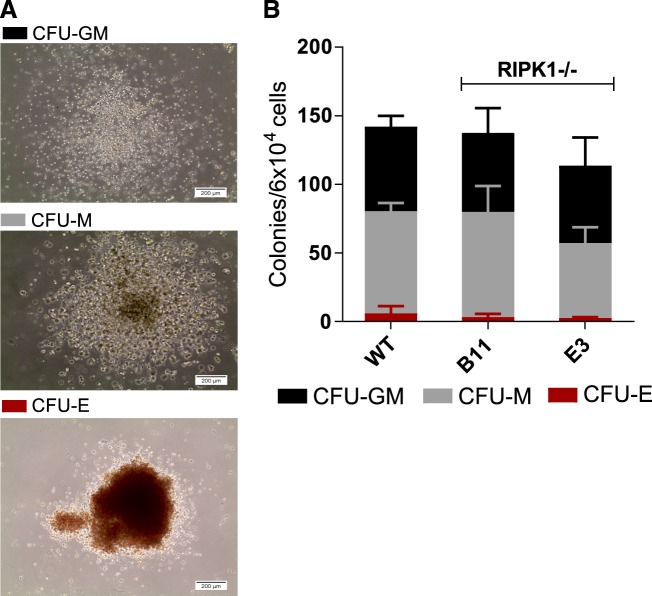
RIPK1^−/−^ iPSCs have normal hematopoietic progenitor numbers. **a** Image of the different colony types. From top to bottom, images show a bright-field image of representative CFU-GM, CFU-M, and CFU-E WT colonies in methylcellulose media at day 14 after methylcellulose plating. **b** Day 14 EBs were dissociated and plated in methylcellulose media, colonies were scored after 14 days of colony growth. Graphs represent mean colony count with SD of WT, RIPK1^−/−^ B11 and RIPK1^−/−^ E3 of three independent experiments (*n* = 3)

The low and transient yield of iPS-MΦ from RIPK1 KO cultures, compared to WT, might have resulted from the loss of hematopoietic progenitor cells or the death of differentiated iPS-MΦ. To distinguish these possibilities, we first assessed the impact of RIPK1 KO on the viability and lineage potential of hematopoietic progenitor cells by performing a colony-forming assay in semisolid media. For this purpose, hiPSC-derived EBs were dissociated after 2 weeks of differentiation, and equal numbers of EB-derived single cells were plated into MethoCult^TM^ H4344. The culture of hematopoietic cell precursors in this medium promotes the formation of erythroid progenitors (CFU-E and BFU-E), granulocyte-macrophage progenitors (CFU-GM, CFU-G, and CFU-M), and multi-potential granulocyte, erythroid, macrophage, and megakaryocyte progenitors (CFU-GEMM) (representative colonies are shown in Fig. 2a). The number and type of CFUs was scored after a further 2 weeks of culture (Fig. 2b). Both RIPK1 KO EBs and WT EBs generated comparable numbers of erythrocyte, granulocyte, and macrophage progenitor colonies of similar size, indicating that RIPK1 is not required for erythro-myeloid differentiation (Fig. 2b).

As the loss of macrophage production occurs after the first wave of production, we tested whether hematopoietic progenitors are lost in RIPK1 KO EBs at later time points. While there was no substantive difference in colony forming potential at 14 and 21 days of differentiation, at day 28, the macrophage colony forming units that were present in the WT were absent in RIPK1 KO EBs, indicative of complete absence of hematopoietic progenitors at this time (Supplementary Figure 5). This indicates that the loss of RIPK1 KO iPS-MΦ occurring from day 21 onwards precedes the loss of hematopoietic progenitors within the EBs (Fig. 1c). Taken together, these results indicate that early hematopoietic progenitors, which are not yet committed to macrophage differentiation, are independent of RIPK1, but that at later time points, during commitment to the macrophage lineage, the absence of RIPK1 affects one or more vital aspect of the cell’s biology.

### KO of RIPK1 induces progressive TNFα-dependent cell death of macrophages

**Fig. 3:**
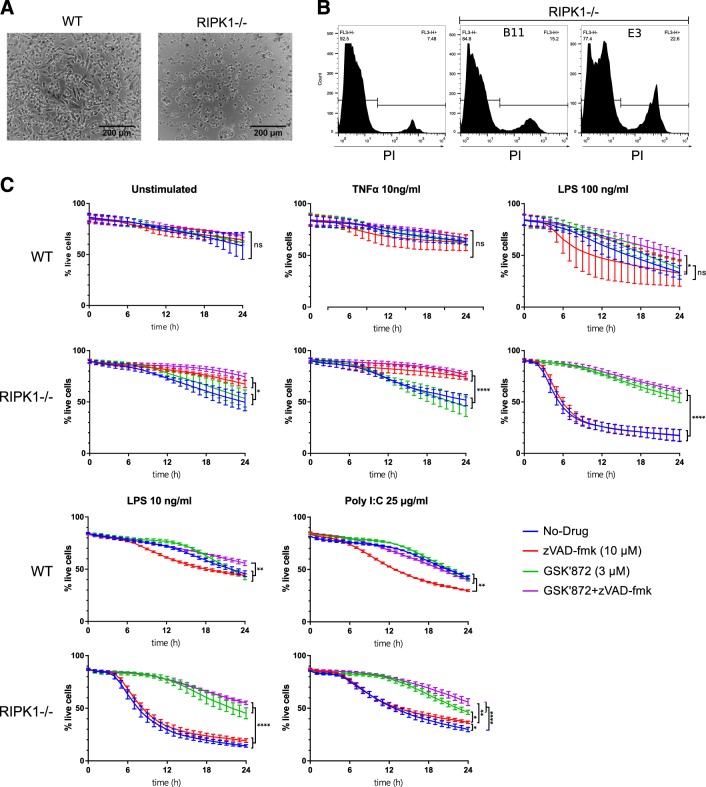
RIPK1^−/−^ macrophages are prone to cell death. LPS and Poly IC induces RIPK3-dependent cell death in RIPK1^−/−^ macrophages. **a** Representative bright field image of unstimulated WT and RIPK1^−/−^ B11 macrophages cultured in macrophage media for 48 h. **b** Representative flow cytometry histograms of unstimulated WT, RIPK1^−/−^ B11 and RIPK1^−/−^ E3 iPSC-derived macrophages stained with PI. **c** iPSC-derived macrophages were harvested and plated in an optical 96-well plate with live/dead stain and imaged for 24 h in presence or absence of LPS (10 or 100 ng/mL), Poly IC (25 µg/mL), TNFα (10 ng/mL), zVAD-fmk (10 µM) and/or GSK872 (3 µM). Percentage of live cells is plotted over time; for untreated, TNFα and LPS 100 ng/mL each point represents the mean cell viability of three independent experiments (*n* = 3). For each experiment the mean viability across three replicate wells was used. For Poly IC and LPS 10 ng/mL each point represents the mean cell viability across three replicate wells (*n* = 1). Error bars denote SD. Statistical comparisons were done on the 24 h timepoint values using one-way ANOVA followed by LSD multiple comparisons test

As RIPK1 is strongly linked to cell death pathways, we investigated whether the decrease in iPS-MΦ number was the result of reduced iPS-MΦ viability. When subjected to the standard protocol for inducing terminal differentiation (see Materials and methods) between 70% and 100% of RIPK1 KO iPS-MΦ died within 48 h, while WT iPS-MΦ differentiated normally and remained viable (Fig. 3a). It was possible that the spontaneous death of RIPK1-KO iPS-MΦ may have resulted from the dysregulation of pathways in which RIPK1 normally transduces inflammatory signals. To test whether TNFα was involved in the cell death of RIPK1 KO iPS-MΦ under normal culture conditions, iPS-MΦ were subjected to the standard protocol for inducing terminal differentiation in presence or absence of 5 µg/mL TNFα neutralizing antibody (Supplementary Figure 6A and B). TNFα neutralizing antibody treatment rescued the cell death phenotype observed previously in RIPK1 KO iPS-MΦ (Fig. 3a). In addition, RIPK1 KO iPS-MΦ treated with TNFα neutralizing antibodies were still viable after 5 days in culture while untreated KO iPS-MΦ were dead (data not shown). This observation suggests that TNFα is involved in the progressive loss of RIPK1 KO iPS- MΦ in culture. In addition, the viability of RIPK1 KO iPS-MΦ before plating was lower compared to WT iPS-MΦ (Fig. 3b).

### KO of RIPK1 sensitizes macrophages to caspase-independent cell death

To further investigate the cell death pathways involved in RIPK1 KO iPSC-MΦ cell death, we stimulated iPS-MΦ with TNFα, LPS or Poly(I:C) to trigger the three most well-characterized inflammatory signaling cascades involving RIPK1 and monitored cell viability by video microscopy over 24 h (Fig. 3c). Unstimulated RIPK1 KO iPS-MΦ died more rapidly than WT iPS-MΦ, an effect which was rescued by inhibition of caspases using zVAD-fmk, but not by inhibition of RIPK3 using GSK-872, indicative of death by apoptosis rather than necroptosis. TNFα produced only a modest effect on the viability of RIPK1 KO iPS-MΦ suggesting that the addition of external TNFα does not change the kinetic of cell death of RIPK1 KO iPS-MΦ observed previously. In contrast, LPS (at both 100 and 10 ng/mL)-induced rapid cell death of RIPK1 KO iPS-MΦ (Fig. 3c), which was counteracted by the RIPK3 inhibitor, GSK-872, but not by inhibition of caspases (Fig. 3c), indicative of necroptosis rather than apoptosis. Note that LPS-induced moderate but variable levels of cell death in the WT (Fig. 3c), but the remaining cells remained viable in prolonged culture (data not shown). As LPS is a known inducer of TNFα in macrophages, we treated iPS-MΦ with 5 µg/mL TNFα neutralizing antibody and stimulated them with 10 ng/mL LPS and monitored cell viability by video microscopy over 12 h (Supplementary Figure 6C). TNFα neutralizing antibody failed to prevent LPS-induced cell death. As a positive control, potency of TNFα neutralizing antibodies was tested on J-Lat as described previously^29,30^. This analysis demonstrated complete neutralization of TNFα (Supplementary Figure 6D, E).

As LPS triggers both the MyD88 and the TRIF pathways^31^, it was not possible to conclude which pathway was responsible for LPS-induced cell death. We therefore stimulated the cells with poly(I:C), which triggers only the TRIF pathway. TRIF is known to mediate formation of secondary endosomal complexes recruiting additional components including RIPK1 and RIPK3^32,33^. Poly(I:C) induced a more modest cell death response than LPS, but one that was similarly dependent on RIPK3 rather than caspases (Fig. 3c). Taken together, these results demonstrate that human RIPK1 KO macrophages are highly sensitive to TRIF-dependent, RIPK3-dependent cell death.

### RIPK1 KO macrophage factories display higher basal level of inflammation and respond strongly to inflammatory signaling

**Fig. 4:**
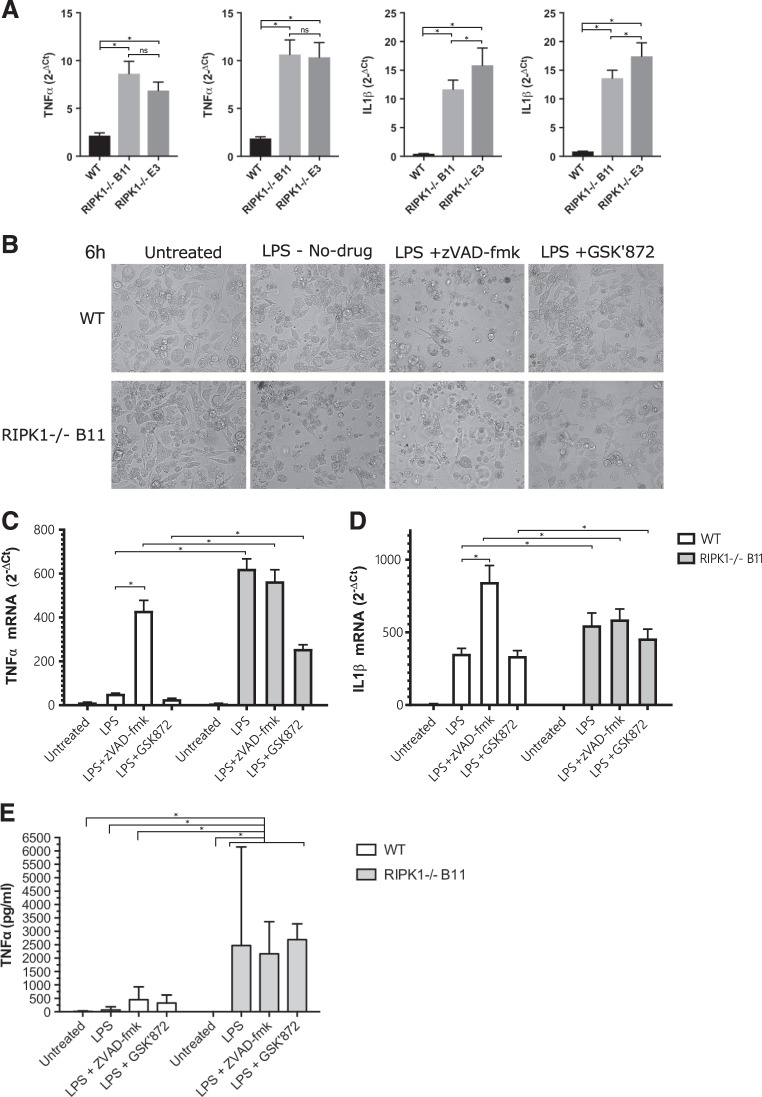
RIPK1^−/−^ iPSC-derived macrophages have higher basal mRNA level of TNFα and IL-1β and higher LPS response. **a** Relative TNFα and IL-1β mRNA normalized to TBP endogenous control (2^−ΔCt^) of freshly harvested, untreated iPSC-derived macrophages. Results from iPS-MΦ of two independent differentiations are shown. **b** Representative images of iPS-MΦ cultured for 6 h in the presence or absence of LPS (100 ng/mL), zVAD-fmk (10 µM) or GSK872 (3 µM). **c**, **d** Relative TNFα and IL-1β mRNA normalized to TBP endogenous control (2^−ΔCt^) of iPS-MΦ cultured for 6 h in the presence or absence of LPS (100 ng/mL), zVAD-fmk (10 µM) or GSK872 (3 µM). Single experiment done in technical triplicate (*n* = 1). **e** TNFα release was measured by ELISA in the supernatant of iPS-MΦ cultured for 3 h in the presence or absence of LPS (10 ng/mL), ZVad-fmk (10 µM) or GSK872 (3 µM). Each condition represents the mean TNFα release across three replicate wells. Error bars denote SD

As RIPK1 KO iPS-MΦ were highly sensitive to death through inflammatory pathways, we hypothesized that the loss of iPS-MΦ during macrophage differentiation was the result of an elevated level of basal inflammation in the cultures. In fact, this view is supported by the protective effect of TNFα neutralizing antibodies against the death of unstimulated RIPK1 KO iPS-MΦ. Therefore, we assessed the levels of TNFα and IL1β mRNA in first harvest macrophages (day 21 post EB formation) using qPCR, and found that they were significantly elevated (Fig. 4a). This indicates that even at an early stage of differentiation, RIPK1 is normally involved in the moderation of macrophage activation and pro-inflammatory pathways in the absence of external pro-inflammatory stimuli.

### TLR4-mediated induction of inflammatory gene expression does not require RIPK1 nor the inhibition of caspase-8

It has recently been suggested that the kinase activity of RIPK1 promotes cell death-independent inflammation in response to TLR4 stimulation and caspase inhibition; conditions typically associated with necrosome formation^14,34,35^. Consequently, we analyzed mRNA levels of TNFα and IL-1β in WT and RIPK1 KO iPS-MΦ before and after exposure to LPS (Fig. 4b–d). In WT iPS-MΦ, as expected, LPS induced the expression of both TNFα and IL-1β mRNA (Fig. 4c, d), and this effect was dramatically enhanced in the presence of the caspase inhibitor, zVAD-fmk, consistent with dependence on necrosome formation, as previously reported^14^. As our results on cell viability, above, indicated that KO of RIPK1 may increase necrosome formation, we hypothesized that it would also enhance the inflammatory response to LPS. Strikingly, the induction of TNFα and IL-1β transcription by LPS in RIPK1 KO iPS-MΦ mimicked that seen in WT iPS-MΦ in the presence of the caspase inhibitor, zVAD-fmk (Fig. 4c, d). The results indicate that RIPK1 and the loss of caspase-8 activity are dispensable for the TLR4-mediated induction of inflammatory gene expression. Since necrosome formation eventually leads to MLKL-dependent membrane breakdown, which may elicit a proinflammatory response in neighboring cells, we analyzed the effect of the RIPK3 inhibitor, GSK-872, on the LPS-response of RIPK1 KO macrophages. Under these conditions, RIPK3 kinase inhibition prevented necrosome-induced cell death (Fig. 3c), and partially prevented the necrosome-associated upregulation of TNFα mRNA (Fig. 4c). We conclude that kinase activity of RIPK3 plays a significant role in driving the necrosome-associated inflammatory response. As a further step, we measured TNFα levels in the extracellular media under conditions minimizing cell death. To achieve this, we treated WT and RIPK1 KO iPS-MΦ with LPS at 10 ng/mL and extracellular TNFα was measured 3 h after treatment. Our live video cell death analysis demonstrated that RIPK1 KO iPS-MΦ triggered necroptosis in response to 10 ng/mL LPS not before 5 h (Fig. 3c). In unstimulated cells, including WT and RIPKO iPS-MΦ, no significant TNFα was detected in collected media (Fig. 4e). In WT iPS-MΦ, exposure to LPS failed to significantly induce TNFα release to the cell media. Notably, TNFα release was highly enhanced in LPS-treated RIPK1 KO iPS-MΦ, which was not prevented by either caspase or RIPK3 inhibition (Fig. 4e). Overall, our results suggest that stimulated RIPK1 KO iPS-MΦ, which are prone to die by necroptosis, elicit a strong inflammatory response along with enhanced TNFα release.

## Discussion

Resident macrophages maintain tissue integrity by clearing debris and dead cells, defending against pathogens, remodeling cells and connective tissue, and secreting trophic factors for both tissue stem cells and their progeny under physiological conditions^36^. However, when their homeostatic capacity is overwhelmed, their inflammatory responses are strongly associated with the pathogenesis of chronic diseases, including type II diabetes, atherosclerosis, age-related neurodegenerative diseases, and chronic inflammatory bowel diseases^37^. Consequently, an understanding of how human tissue macrophages fine-tune their inflammatory response to external signals is required for rational approaches to treating these increasingly prevalent diseases, without compromising their vital tissue defence and homeostatic functions. Studies, largely on human cancer cell lines in vitro and in transgenic mouse models, have revealed a complex web of regulatory pathways that integrate the responses of cells to inflammatory cytokines, such as TNF and to pathogen-associated molecular patterns (PAMPs) such as LPS^7,12,16,17,21^. In these reports, RIPK1 is found to play an important role; sometimes promoting and sometimes inhibiting the effector functions of caspase-8 and RIPK3, depending on circumstance

**Fig. 5:**
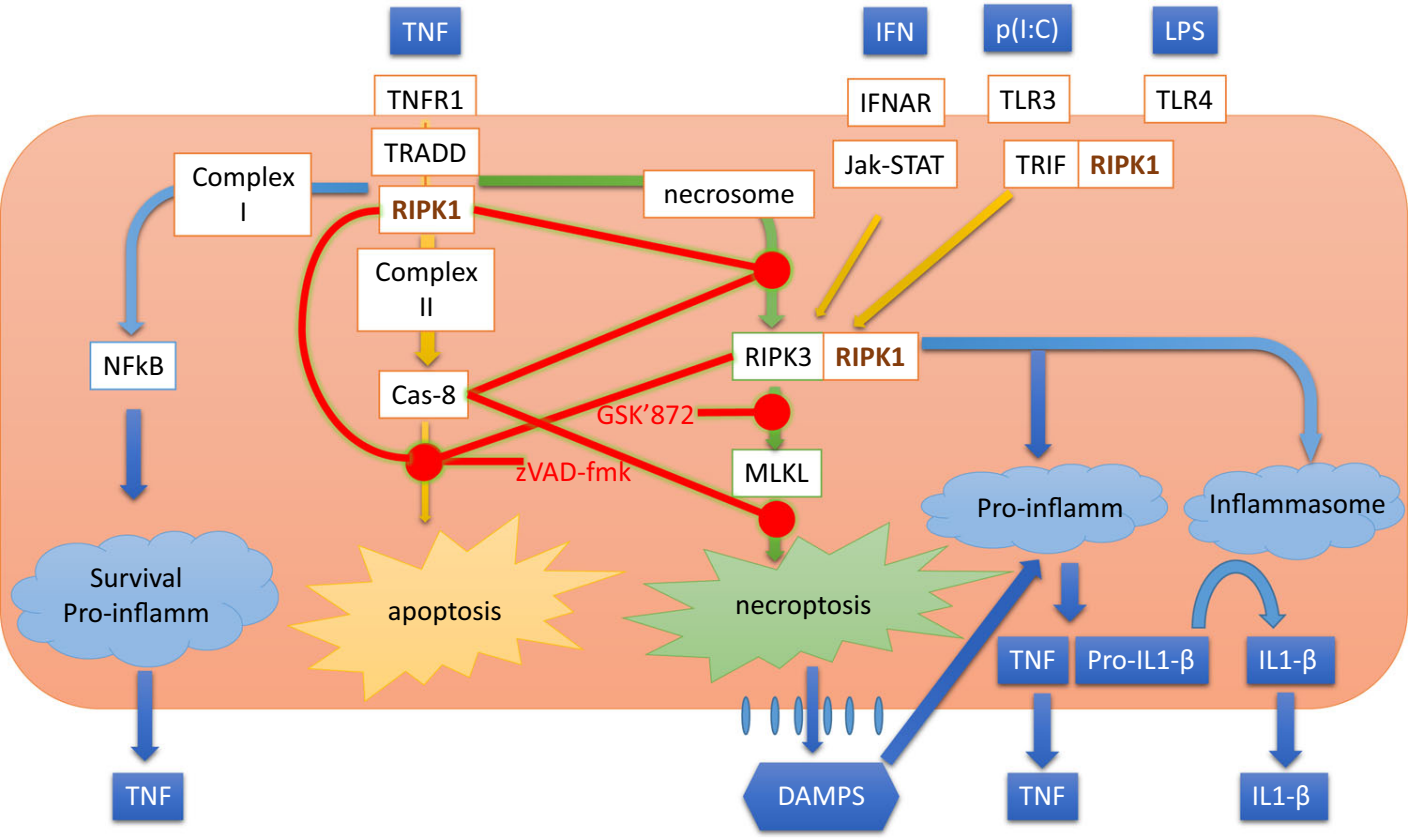
Scheme illustrating potential mechanisms by which RIPK1 regulates inflammatory and cell death pathways in human macrophages. Main receptors engaged to RIPK1 signaling are TNFR1, TLR3 and TLR4 (via TRIF pathway) and interferon receptors. TNFR stimulation recruits TRADD and RIPK1 thus leading to the formation of membrane bound complex I and subsequent NF-kB activation. At this step, NF-kB triggers transcription of proinflammatory (i.e. TNF and pro-IL1β) and survival factors (i.e. cFLIP; not shown). Deubiquitination of Complex I induces the formation of Complex II, which may induce apoptosis through activation of caspase-8 (Cas-8). However, under conditions of caspase-8 inhibition, which can be induced by the pan-caspase inhibitor zVAD-fmk (zVAD), a cell death-related platform called necrosome is formed. Necrosome formation is also associated to stimulation of TLR3, TLR4, and IFNAR through interaction and further oligomerization of RIPK1 and RIPK3, a process also requiring reduced caspase-8 activity. Necrosome formation induces the phosphorylation of MLKL, which then disrupts membrane integrity to cause necroptosis. This process can be prevented by kinase inhibitors of RIPK3, like GSK´872. Necroptosis is accompanied by concomitant release of danger-associated molecular patterns (DAMPS), which may amplify the inflammatory response through stimulation of pattern recognition receptors, including the ones depicted in the figure. In absence of RIPK1, necrosome formation (RIPK3 oligomerization) is achieved without the need of caspase-8 inhibition. Necrosome formation is inherently associated to strong inflammatory response, which can be partially prevented by RIPK3 kinase inhibition

In order to investigate the relative contribution of RIPK1 to inflammatory and cell death pathways in normal human macrophage development and function, in this study we have generated KO of RIPK1 in human iPS cells, and used a well-established model of iPS-MΦ differentiation. We show that RIPK1 is not required for erythro-myeloid differentiation. However, soon after the initiation of differentiation, in the absence of RIPK1, hematopoietic progenitors are lost, and unstimulated differentiated iPS-MΦ switch to a primed, proinflammatory phenotype, and then tend to die. As myeloid precursors become committed to the macrophage differentiation pathway, they acquire potent, lineage-specific response pathways that need to be held under control in order to prevent self-inflicted damage in the absence of an appropriate external stimulus. Our results suggest that RIPK1 might be critical in maintaining tonic control of macrophage activation pathways, allowing the cells to respond rapidly to stimuli from an active but sub-critical level. In this regard it resembles the phenomenon of tonic signaling in lymphocytes^38^. Supporting this view, we provide evidence that in the absence of RIPK1, unstimulated differentiated iPS-MΦ die by intrinsic TNFα-dependent apoptosis.

Our results show that activation of RIPK1 KO iPS-MΦ by TLR ligands triggers a robust induction of TNF mRNA transcription and commits them to die by necroptosis. The necroptotic nature of the TLR4-induced cell death in RIPK1 KO macrophages was confirmed by using GSK-872, a specific RIPK3 inhibitor, that prevented cell death. TLR3 stimulation by poly(I:C) likewise resulted in RIPK3-dependent, caspase-independent cell death, confirming the ability of TRIF to trigger RIPK3 activation without the need for RIPK1 recruitment.

The first evidence demonstrating a scaffolding role of RIPK1 in preventing necroptosis came from studies using transgenic mice showing that loss of RIPK3 protects RIPK1/caspase-8 KO mice, which die shortly after birth^16,39,40^. Further studies have shown the ability of RIPK1 to prevent necroptosis in murine cells^11,12^. Our study demonstrates that RIPK1 plays a pro-survival role against an autocrine apoptotic TNFα tonic signal in unstimulated macrophages and against necroptosis in TLR-stimulated human macrophages. In some settings, it has been established that necroptosis requires RIPK1-kinase activity^41,42^. Our data are consistent with the view that this kinase activity counter-balances the scaffolding role of RIPK1 in preventing necroptosis in activated tissue-resident macrophages.

Our experimental system, using human hiPSCs lacking RIPK1, has enabled us to ascertain the potentially divergent roles of RIPK1 in regulating cell death and inflammation, respectively (Fig. 5). As a first step, we analyzed the mRNA levels of two key proinflammatory cytokines orchestrating inflammation, TNFα and IL-1β, in unstimulated human WT and RIPK1 KO macrophages^7^. Strikingly, the basal transcription of both genes was strongly elevated in macrophages lacking RIPK1, consistent with a chronic primed proinflammatory phenotype. It is well established that exposure of primed macrophages to DAMPs make them extremely reactive, secreting large amounts of proinflammatory molecules^43^. Chronic proinflammatory activation of RIPK1 KO human macrophages could result from a vicious cycle of necroptosis, DAMP release and priming of bystander cells. However, the necrosome may elicit an intrinsic MLKL-independent proinflamamatory response through engagement of the NLRP3 inflammasome to activate IL1β^44^. RIPK3 has been also shown to facilitate the production of cytokines independent on their role in necroptosis in response to combined IAP deletion, inhibition by Smac mimetic treatment or even XIAP deletion^45,46^. More recently, critical roles for the kinase activities of RIPK1 and RIPK3 in LPS-induced expression of inflammatory cytokines in bone marrow-derived macrophages have been revealed^14,35^. Consequently, there is strong evidence supporting the view that necroptosis-driven inflammatory response does not exclusively rely on DAMP release.

Accordingly, we sought to analyze the role of RIPK1 in regulating inflammation under conditions of necrosome formation. In confirmation of earlier findings in WT cells, caspase inhibition increased TNFα and IL-1β mRNA levels in LPS-stimulated iPS-MΦ, a process that has been suggested to be regulated by RIPK1 kinase activity^14^. However, if RIPK1 were a requirement for the necrosome-dependent inflammatory pathways, inflammatory cytokine expression should be attenuated in LPS-treated, caspase-inhibited RIPK1 KO compared to WT iPS-MΦ, whereas they showed comparable levels. Intriguingly, RIPK3 kinase inhibition partially prevented the upregulation of TNF transcription by LPS in RIPK1 KO macrophages. We conclude that RIPK1 is dispensable, while RIPK3 is indispensable, for the necrosome-dependent, cell-intrinsic inflammatory response.

Our findings suggest that RIPK1 is not only dispensable for necrosome-dependent inflammatory pathways in human macrophages, but plays a key role in maintaining them in a tonic state that prevents inappropriate, cell intrinsic TNFα-dependent apoptosis in the absence of appropriate external stimuli, so that these very potent defences are not deployed prematurely, resulting in self-harm. Elucidating these RIPK1-dependent regulatory pathways may open new therapeutic strategies against chronic pathological inflammatory diseases in which proinflammatory priming of macrophages is a distinctive feature.

## Materials and methods

### hiPSC culture

The previously characterized human iPSC line OX1-61 (alternative name SFC841-03-01)^47^, reprogrammed using non-integrating Cytotune Sendai virus reprogramming kit (Invitrogen), was the target line for all gene editing in this study. The line was originally derived from a healthy donor recruited through the Oxford Parkinson’s Disease Centre having given signed informed consent, which included derivation of hiPSC lines from skin biopsies (Ethics Committee that specifically approved this part of the study, National Health Service, Health Research Authority, NRES Committee South Central, Berkshire, UK, REC 10/H0505/71). All experiments were performed in accordance with UK guidelines and regulations and as set out in the REC. The line has been deposited in the European Bank for iPS cells, EBiSC, https://cells.ebisc.org/STBCi044-A. Low-passage, QC’ed stocks of iPSCs were thawed and plated on hESC qualified matrigel-coated plates (Scientific Laboratory Supplies 354277) and cultured in mTeSR^TM^1 (Stemcell^TM^ technologies) media, passaging routinely with 0.5 mM EDTA^48^, or when necessary with TrypLE (Thermofisher) and supplementing the replating medium with 10 µmol/L Rho-kinase inhibitor Y-27632 (Abcam).

### CRISPR-Cas9 gene editing

The CRISPR-Cas9 vectors used in this study were based on the dual Cas9- and guide RNA (gRNA)-, puromycin-resistance gene-expressing, pSpCas9n(BB)-2A-Puro (pX462) vector^49^ (a gift from Feng Zhang: Addgene plasmid #48141). Vectors were cloned as previously described^49^ using oligonucleotides 5′-CACCGAGTTTGCTCCACATCTTAA-3′ and 5′-AAACTTAAGATGTGGAGCAAACTC-3′ with pX462 to create pX462-gRIPK1t; oligonucleotides. Transfection of the plasmids was performed by electroporation on 2 × 10^6^ feeder-free iPSCs in single-cell suspension (Neon^®^ transfection system, Invitrogen), as previously described^50^. Cells were subsequently selected for puromycin resistance (0.4 µg/mL; MP Biomedicals) for 48 h, after which the surviving cells were plated on mitotically inactivated mouse embryonic fibroblast feeder cells (MEFs)^51,52^ in hESC medium (KO-DMEM, 2 mmol/L l-glutamine, 100 mmol/L non-essential amino acids, 20% serum replacement, and 8 ng/mL basic fibroblastic growth factor (FGF2)), supplemented with 10 µmol/L Y-27632 on the day of passage. The single cell colonies were picked manually onto Matrigel-coated 96-well plates in mTeSR^TM^1 and expanded for 7 days. Individual clones were then pre-screened by high-resolution melt analysis (HRM) on a StepOnePlus^TM^ Real-Time PCR System (ThermoFisher), using the primers 5′-CAAACAATCCCAGTGGCTCAA-3′ and 5′-GTAGTAGAGGGTGCCGCCATT-3′. Potential KO clones were then sequenced and analyzed for insertions and/or deletions. The selected clones were subsequently expanded and characterized as previously described^47^ to confirm expression of pluripotency proteins TRA-1-60 and NANOG, and SNP analysis was conducted to confirm normal karyotype and for cell line tracking, using Illumina OmniExpress24 array (700,000 markers).

### Macrophage differentiation from iPSCs

EBs were generated as previously described^25,26^, with minor modifications as follows. iPSC in single cell suspension were centrifuged in 96-well ultra-low adherence plates (Costar 7007) at 1.25 × 10^5^ cells/mL in EB media (mTeSR^TM^1 (StemcellTM technologies), 50 ng/mL BMP-4 (GIBCO PHC9534), 20 ng/mL SCF (Miltenyi Biotec Ltd), 50 ng/mL VEGF (GIBCO-PHC9394)) supplemented with 10 µmol/L Y-27632 on the day of EB formation. Cells were fed with EB media ± 50 µL for 4 days. After 4 days, the differentiated EBs were transferred into a six-well tissue culture plate at a ratio of 8 EBs/well in monocyte differentiation media (X-VIVO^TM^-15 (Lonza), 100 ng/mL M-CSF (Invitrogen), 25 ng/mL IL-3 (R&D), 2 mM glutamax (Invitrogen), and 0.055 mM 2-mercaptoethanol (Invitrogen). From weeks 3 to 5, the non-adherent iPS-MΦ produced were harvested every week and counted using NC-3000 Viability and Cell Count Assays (Chemometec) according to manufacturer’s instructions.

### iPS-MΦ terminal differentiation

For terminal differentiation of iPS-MΦ, freshly harvested non-adherent iPS-MΦ were plated in macrophage differentiation media (X-VIVO™-15 (Lonza), supplemented with 100 ng/mL M-CSF (Invitrogen), 2 mM glutamax (Invitrogen), 100 U/mL penicillin, and 100 mg/μL streptomycin (Invitrogen), and cultured for an additional week.

### Embryoid body dissociation and colony forming assay

Day 14 EBs were dissociated by treatment with Accumax™ Solution (Sigma) at 37 °C followed by mechanical dissociation (as described previously^27^). An additional 10-min incubation step was added for the day 21 and day 28 EBs. The resulting cell suspension was filtered and 3 × 10^4^ cells plated in MethoCult^TM^H4344 (STEMCELL technologies) semisolid media. On day 14 post-dissociation, colonies were classified and counted based on their morphology.

### Quantification of cell death by live microscopy

hiPS-MΦ were resuspended in macrophage media supplemented with 20 µL/mL ReadyProbes Cell Viability Kit, Blue/Green (ThermoFisher-R37609). hiPSC-MΦ were then plated at 3 × 10^4^ cells per well in a clear bottom 96-well plate and treated with: 10 ng/mL TNFα (Peprotech) 10 or 100 ng/mL LPS (Sigma), 25 µg/mL poly(I:C) (Sigma), 10 µM zVAD-fmk (BD Pharmingen^TM^-550377) and/or 3 µM GSK872 (BioVision-2673-5). Cells were imaged every hour for 24 h in an EVOS automated microscope (Thermofisher) in a humidified 37 C, 5% CO_2_ incubation chamber. Number of green (dead) and live (blue) nuclei was calculated using imageJ. Percentage of viable cells over time was defined as the ratio between live and dead cells.

### Flow cytometry staining and antibodies

Harvested macrophages were pelleted at 400×*g* for 5 min and washed once with PBS before being resuspended in 100 μL of FACS buffer (PBS + 10 μg/mL human serum IgG + 1% fetal bovine serum (FBS)). Cells were stained in FACS buffer + antibody (dilution 1:100) for 45 min at 4 °C or propidium iodide (PI) stained at 1 µg/mL for 15 min at RT. PI Stained cells were washed PBS and analyzed using a FACSCalibur flow cytometer (BD) without fixation. Antibody stained cells where washed using PBS and resuspended in 2% formaldehyde before being analyzed using a FACSCalibur flow cytometer (BD). The following antibodies have been used in this study: α-CD14-FITC (antibody (MEM-15; Immunotools), Mouse IgG1-FITC isotype (PPV-06; ImmunoTools), α-CD16-APC (LNK16; Immunotools), Mouse IgG1-APC isotype (PPV-06; ImmunoTools), α-CD11b-APC (ICRF44; BioLegend), Mouse IgG1-APC Isotype (MOPC-21; BioLegend). iPSC lines were stained for TRA-1-60 (1.5 mg/mL; α-TRA-1-60-AlexaFluor R488; Biolegend) and NANOG (0.3 mg/mL; α-NANOG-AlexaFluor R647; Cell Signaling Technologies) as previously described^47^.

### RNA extraction, reverse transcription and quantitative polymerase chain reaction

Freshly harvested iPSC-MΦ were either pelleted and lysed directly or plated at 7 × 10^5^ cells/well in a 12-well in macrophage media and cultured for 6 h in presence or absence of 100 ng/mL LPS, 10 µM zVAD-fmk and/or 3 µM GSK before being lysed in the plate. Cells were lysed using RLT buffer (QIAGEN) supplemented with 10 µL β-ME. RNA extraction was performed using the RNeasy^®^ kit (QIAGEN) according to manufacturer’s protocol. Potential DNA contamination was removed by adding a step of Ambion^®^ TURBO DNA-free^®^ according to manufacturer’s protocol (Life technologies). Reverse transcription was performed using the High capacity RNA-to-cDNA kit (Applied Biosystems™) according to manufacturer’s protocol. qPCR was performed using Brilliant III SYBR^®^ (Agilent) on the Applied Biosystems^®^ StepOnePlus™ Real-Time *PCR* System. The Following primers were used:

**Table:** 

Target	Forward primer (5′–3′)	Reverse primer (5′–3′)
TATABox protein	GAACCACGGCACTGATTTTC	CCCCACCATGTTCTGAATCT
RIPK1	TTACATGGAAAAGGCGTGATACA	AGGTCTGCGATCTTAATGTGGA
TNFα	TGTTGTAGCAAACCCTCAAGC	TATCTCTCAGCTCCACGCCA
IL1-β	AAAGCTTGGTGATGTCTGGTC	GGACATGGAGAACACCACTTG

### Western blot

Four-day old EBs or undifferentiated iPSCs were lysed in RIPA buffer (Sigma) for 30 min on ice. Lysates were centrifuged at 17,000*×g* for 15 min at 4 °C and clarified supernatant protein concentration was quantified using Pierce^®^ BCA protein assay kit (Thermo Fisher Scientific) and 25 μg of protein was denatured in NuPAGE LDS Sample Buffer and NuPAGE™ Sample Reducing Agent by heating to 70 °C for 5 min. Proteins were run on a NuPAGE 4–12% Bis–Tris Gel (Life technologies), and transferred on a nitrocellulose membrane (Amersham Protran). The membrane was stained overnight at 4 °C in PBS + 5% BSA + 0.05% Tween 20 with primary antibodies: 0.5 µg/mL of mouse monoclonal RIPK1 antibody (MAB3585-R&D) and 0.1 µg/mL of polyclonal rabbit Anti-α-Actin-1 antibody (A00885-GenScript). Secondary antibody staining was done using Goat anti-Mouse IgG (H + L) Secondary Antibody, DyLight 800 4X PEG and Goat anti-Rabbit IgG (H + L) Secondary Antibody, DyLight 680 at 1:10,000 for 2 h at RT. Blot was scanned using a LI-COR Odyssey scanner.

### TNFα ELISA

Freshly harvested iPS-MΦ were plated at 3 × 10^4^ cells per well in a 96-well plate in 100 µL macrophage media and treated with 10 ng/mL LPS (Sigma), 10 µM zVAD-fmk (BD Pharmingen^TM^-550377) and/or 3 µM GSK872 (BioVision-2673-5). Supernatant was collected after 3 h and stored at −80 °C before ELISA. Human TNF alpha Uncoated ELISA (Invitrogen-88-7346-22) was performed according to manufacturer’s instructions, media samples were diluted 1:50 and 100 µL was used.

### TNFα blocking experiments

For quantification of TNFα involvement in the cell death of RIPK1 KO iPS-MΦ under normal culture conditions, iPS-MΦ were plated at 3 × 104 cells per 96-well and subjected to the standard protocol for inducing terminal differentiation in presence or absence of 5 µg/mL TNFα neutralizing antibody. 48 h after plating cells, images were taken and total number of cells per field was quantified manually on ImageJ. For live quantification of cell death, iPS-MΦ were harvested and plated in a four chamber ibidi µ-Dish 35 mm dish in MΦ-media containing 10 µg/mL PI (Invitrogen-P3566) and preincubated for 2 h with 5 µg/mL TNFα neutralizing antibody (R&D-MAB2101R). iPS-MΦ were imaged for 12 h, one image per 15 min in presence or absence of LPS (10 ng/mL) in a Nikon BioStation iMq. Five fields were acquired per condition. Total number of starting cells per field were counted manually on imageJ and number of PI positive nuclei were counted automatically for each timepoint using an imageJ macro. For J.Lat 10.6 cells reactivation, 10^5^ J.Lat 10.6 were cultured in RPMI + 10% FBS in a round 96-well plate and preincubated for 2 h with or without 5 µg/mL TNFα neutralizing antibody (R&D-MAB2101R) after which they were stimulated with increasing concentrations of recombinant human TNFα (Sigma-H8916). After 24 h, cells were fixed for 10 min in 4% paraformaldehyde and percentage of GFP+ cells was quantified by flow cytometry on a BD FACSCanto II.

## Electronic supplementary material

Supplementary Figure 1

Supplementary Figure 2

Supplementary Figure 3

Supplementary Figure 4

Supplementary Figure 5

Supplementary Figure 6
